# A Cluster Randomized Controlled Trial of a Lay Health Worker Intervention to Increase Healthy Eating and Physical Activity Among Vietnamese Americans

**DOI:** 10.5888/pcd17.190353

**Published:** 2020-04-30

**Authors:** Jane Jih, Susan L. Stewart, Thien-Nhien Luong, Tung T. Nguyen, Stephen J. McPhee, Bang H. Nguyen

**Affiliations:** 1 Division of General Internal Medicine, University of California, San Francisco; 2 Multiethnic Health Equity Research Center, University of California, San Francisco; 3 Asian American Research Center on Health, San Francisco, California; 4 Department of Public Health Sciences, University of California, Davis; 5 Vietnamese Reach for Health Coalition, Fremont, California; 6 Research Department, Cancer Prevention Institute of California, Fremont, California

## Abstract

**Introduction:**

Americans have low levels of knowledge of and adherence to recommendations for healthy eating of fruits and vegetables and for physical activity (HEPA). We conducted a cluster randomized controlled trial of a lay health worker intervention to increase HEPA among Vietnamese Americans.

**Methods:**

We randomized 64 lay health workers to 2 intervention arms. Each lay health worker recruited 10 participants aged 50 to 74. From 2008 to 2013, using flip charts, lay health workers led 2 educational sessions on HEPA (intervention) or colorectal cancer (comparison). We assessed HEPA knowledge and self-reported behaviors by preintervention and postintervention surveys 6 months apart.

**Results:**

Of the 640 participants, 50.0% were female, 38.4% had lived in the United States for 10 years or fewer, and 71.4% reported limited English proficiency. Knowledge of the recommended intake of fruits and vegetables (≥5 servings daily) increased from 2.6% to 60.5% in the intervention group (n = 311) and from 2.9% to 6.7% in the comparison group (n = 316) (intervention vs comparison change, *P* < .001). Knowledge of the physical activity recommendation (≥150 minutes weekly) increased from 2.6% to 62.4% among intervention participants and from 1.0% to 2.5% among comparison participants (*P* < .001). Consumption of 5 or more daily servings of fruits and vegetables increased more in the intervention group (8.4% to 62.1%) than in the comparison group (5.1% to 12.7%) (*P* < .001). Participants reporting 150 minutes or more of physical activity weekly increased from 28.9% to 54.0% in the intervention group and from 38.0% to 46.8% in the comparison group (intervention vs comparison change, *P* = .001).

**Conclusion:**

A lay health worker intervention increased both healthy eating and physical activity knowledge and self-reported behaviors among older Vietnamese Americans.

**Summary**

**What is already known on this topic?**

Although disparities in healthy eating and physical activity exist between Asian Americans, including Vietnamese Americans, and the non-Hispanic white population, few intervention studies have been conducted among them.

**What is added by this report?**

A lay health worker intervention was efficacious in increasing both healthy eating and physical activity knowledge and self-reported behaviors among Vietnamese Americans.

**What are the implications for public health practice?**

A lay health worker–led intervention was a feasible, accessible, and efficacious approach to address disparities in healthy eating and physical activity knowledge and behaviors and to promote health equity among Vietnamese Americans.

## Introduction

Greater fruit and vegetable consumption is associated with a lower risk of myocardial infarction, stroke, type 2 diabetes mellitus, some cancers, and weight gain (1). In addition, more physically active adults have lower rates of all-cause mortality, coronary heart disease, hypertension, stroke, type 2 diabetes mellitus, metabolic syndrome, cancer, and depression (2). Physically active adults have a higher level of cardiorespiratory and muscular fitness, a healthier body mass and composition, and better quality of sleep and health-related quality of life (2). Significant disparities in healthy eating and physical activity (HEPA) exist between the non-Hispanic white population and many racial/ethnic groups in the United States (3), including Asian Americans, among whom few intervention studies have been conducted.

Vietnamese Americans are the fourth-fastest growing Asian group in the United States (4,5); the population of Vietnamese Americans was more than 1.5 million in 2010 (5). Socioeconomic and health disparities exist between Vietnamese Americans and the non-Hispanic white population. Most Vietnamese Americans were born outside the United States (68%), speak Vietnamese at home (88%), and have limited English proficiency (60%) (5). Fourteen percent live in poverty, and only 24% have completed college (5).

A few studies on the diet and physical activity of Vietnamese Americans (6–11) have been published. Although 2005 and 2010 dietary guidelines for Americans recommended intake of least 5 servings of fruits and vegetables daily (1,12), a study published in 1995 reported that Vietnamese Americans consumed only 3.5 servings of fruits and vegetables per day (8). Since 2008, US physical activity guidelines have recommended at least 150 minutes of moderate or vigorous physical activity weekly (2,13). However, a greater percentage of Vietnamese Americans (40%) than non-Hispanic white Americans (12%) surveyed from 2002 through 2005 reported no moderate or vigorous physical activity (11). Compared with non-Hispanic white Americans, Vietnamese Americans had less knowledge of cardiovascular disease symptoms and Vietnamese men were at a greater risk of hemorrhagic stroke (11,14).

Few randomized controlled trials of healthy eating and physical activity interventions have been conducted among Asian Americans in general or among Vietnamese Americans in particular (7,9). A promising approach to promoting healthy behavior among underserved, immigrant, and minority groups is to engage culturally and linguistically concordant lay health workers in interventions (15). Lay health worker interventions focused on breast, cervical, and colorectal cancer screening significantly improved cancer screening behaviors among Asian Americans, including Vietnamese Americans (11,16–21). We conducted a lay health worker intervention to increase HEPA among Vietnamese Americans. The objective of this study was to describe the effectiveness of the intervention in improving HEPA knowledge and self-reported behaviors.

## Methods

More than one-third of Vietnamese Americans live in California (4). We chose Santa Clara County, California, as the study site because it has a large Vietnamese American population in close proximity to the research team. We formed a coalition of academic research institutions and community organizations, leaders, and members to design and implement the study as well as to analyze, interpret, and disseminate findings. The lay health worker activities were carried out in the study area by 4 community-based organizations in the coalition. These activities took place from 2008 to 2013 and received institutional review board approval from the Cancer Prevention Institute of California and the University of California, San Francisco.

### Study design, randomization, and recruitment

We used a cluster randomized controlled trial study design; details on study design, randomization and recruitment are provided elsewhere (22). Briefly, lay health workers and their recruited participants were randomized to 2 study arms. Intervention-arm lay health workers led 2 educational sessions about HEPA. Comparison-arm lay health workers led 2 educational sessions about colorectal cancer screening (CRC). The efficacy of the intervention was measured through preintervention and postintervention surveys administered by trained, bilingual interviewers.

Each community-based organization had a half-time coordinator for the lay health worker intervention. After training by the research staff, each coordinator recruited 16 lay health workers with equal numbers of men and women from the organization’s client base and their social networks (family, friends, and referrals). Eligibility criteria for lay health workers were 1) self-identification as Vietnamese or Vietnamese American, 2) age 50 to 74, 3) understanding of spoken and written Vietnamese, and 4) living and intending to stay in the study area for study duration. Research staff members randomized lay health workers and their recruited participants together as clusters to either the intervention or the comparison arm in a 1-to-1 ratio.

Inclusion criteria for participants were the same as the lay health worker eligibility criteria, plus never having had CRC screening (fecal occult blood test, sigmoidoscopy, or colonoscopy). The single exclusion criterion was living in the same household as another study participant.

The lay health workers and participants in this HEPA intervention study were the same as those in the CRC intervention study; the only difference was that the participants in the comparison arm of the CRC study were in the intervention arm of the HEPA study (and vice versa) (22). The sample size of 640 participants provided 80% power to detect a 20 percentage-point difference (eg, 40% vs 20%) between the study arms in the proportion screened for CRC in the CRC intervention at postintervention among both men and women at the .05 level (2-sided), assuming an intracluster correlation coefficient of 0.05, an attrition rate of 0.05, and a cluster size of 10 participants per lay health worker.

### Theoretical framework

The theoretical framework for the lay health worker intervention was the Pathways framework and the Diffusion of Innovations theory, as described previously (22). According to the Pathways framework, knowledge, attitudes, and beliefs are pathways to behavioral changes. The intervention-arm lay health workers delivered educational sessions, health educational materials, and follow-up support services to improve participants’ HEPA knowledge, attitudes, and behaviors. According to the Diffusion of Innovations theory, lay health workers were innovators or change agents because they asked participants to eat more fruits and vegetables that few had eaten and to do more moderate and/or vigorous physical activities that few had previously done at the recommended levels.

### Health educational materials and formative research

We developed the educational materials “from scratch” to develop a culturally and linguistically appropriate communication mechanism, content, and illustration (23). We chose the flip chart format as a teaching tool for lay health workers to make presentations in small group sessions because it does not require lay health workers to be proficient in the use of a technological device. We developed the HEPA flip chart in Vietnamese to connect directly to the target audience by using their vernacular and their colloquial and idiomatic expressions.

A team of bilingual and bicultural staff and consultants drafted the flip chart content to illustrate healthy dietary items and physical activities. The content included information on foods commonly eaten (eg, serving size) and on physical activities commonly done by Vietnamese Americans. A bilingual and bicultural professional graphic designer produced a flip chart using images of foods, including fruits and vegetables, and illustrated the text with Vietnamese American models consuming healthy food and engaging in physical activities. A bilingual and bicultural registered dietician reviewed the content for scientific accuracy, and trained research staff members on serving sizes. The dietary recommendations followed the *Dietary Guidelines for Americans, 2005,* for the general population, and the physical activity recommendations followed the *Physical Activity Guidelines for Americans, 2008,* for adults (12,13). In developing the flip charts, we also conducted 8 cognitive interviews of community members who otherwise did not participate in the research (24).

**Figure 1:**
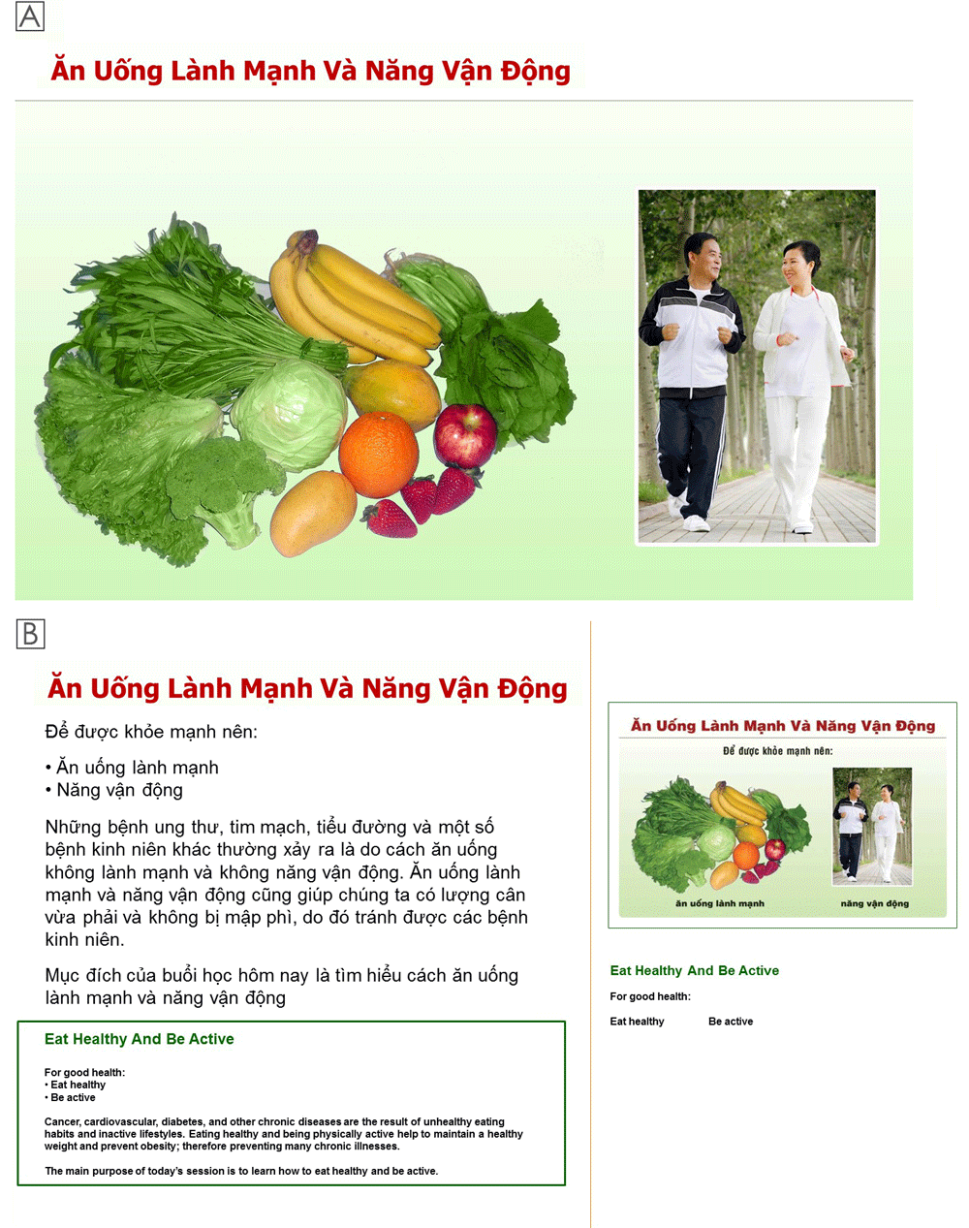
Culturally and linguistically appropriate educational materials developed for delivery by lay health workers to Vietnamese Americans aged 50 to 74 in an intervention designed to increase healthy eating and physical activity, Santa Clara County, California, 2008–2013. A, Participant-facing page of a flip chart. B, Lay health worker–facing page.

The HEPA flip chart was printed on 18-by-12-inch glossy, heavy stock; spiral bound, it could be set up as a tent on a table to be viewed by the small group. The page facing participants contained educational messages in Vietnamese with graphics, photographs, and illustrations in full color for the participants to view and follow the oral presentation. The page facing the lay health worker contained teaching points in Vietnamese and a small image of the front page with an English translation to aid the lay health workers in delivering the presentation (Figure 1). We also developed a booklet with identical messages and images as on the front of the flip chart, except that the booklet also contained the same messages in English at the bottom of each page. The booklet was distributed to participants at the educational sessions for them to take home to reinforce the messages presented at the sessions. We also developed a CRC screening flip chart and booklet for the comparison group by using the same formative research methodology.

Lay health workers attended trainings, recruited participants, delivered 2 educational sessions, distributed health educational booklets, and provided telephone or in-person follow-up support to participants. Each lay health worker received $1,200 to perform these functions. During 2 days (for a total of 12 hours), research staff members and agency coordinators trained the lay health workers in each study arm separately on the different health topics. All lay health workers received training about recruitment of participants, outreach approaches and procedures, and organization and facilitation of educational sessions. The intervention group lay health workers were educated about HEPA, including information on serving sizes; the comparison group lay health workers learned about CRC screening. Lay health workers then practiced delivering their presentations using the flip charts. We gave a reference manual to each lay health worker.

After being trained, each lay health worker recruited 10 participants of their own sex. Research staff obtained informed consent from participants by telephone. Intervention lay health workers used the portable flipchart in the first educational group session to educate the participants about HEPA and to recommend HEPA behaviors that met recommended guidelines. Food replicas in appropriate serving sizes were used as teaching aids and handed to participants to examine at the group session. At the end of this session, the lay health workers distributed the HEPA booklets. In the second session that took place 2 to 3 months after the first, each lay health worker identified participants who had not met recommended HEPA behaviors, identified barriers to HEPA behavior change, and provided suggestions and support to overcome these barriers. Each session took 1 to 2 hours. After each session, the intervention lay health worker made follow-up telephone calls or in-person visits to participants to remind them to follow HEPA recommendations. We did not pay participants for intervention activities.

### Measures

Before the first educational session, research staff members conducted a telephone survey of all participants to collect baseline data. Research staff members conducted a second telephone survey 6 months after the first educational session (approximately 3 or 4 months after the second session) to allow intervention group participants sufficient opportunity to follow HEPA recommendations. The outcome variables were HEPA knowledge and self-reported behaviors. We used 2 items to assess HEPA knowledge: 1) knowing the recommended minimum daily number of fruit and vegetable servings (5 per day) and 2) knowing the recommended minimum weekly amount of moderate and/or vigorous physical activity (150 minutes per week). For these knowledge measures, participants had an option to provide a numeric response, state “do not know” or “unsure,” or refuse to respond. We used 2 items to assess HEPA self-reported behaviors: 1) the number of servings of fruits and vegetables eaten during the previous day and 2) the duration of at least moderate-intensity physical activity during the previous week. We coded each behavior as meeting or not meeting the guideline. After defining a serving size of fruits and vegetables with an example (“one serving of vegetables includes a half–medium-sized rice bowl of boiled greens”), research staff members separately asked for a numeric response for servings of fruits and vegetables eaten during the previous day. After defining moderate and vigorous physical activity, research staff members asked participants whether they participated in that intensity of physical activity and on how many days of the week and for what duration (hour[s] and minutes). We calculated the number of minutes of at least moderate-intensity physical activity during the previous week. Participants who were unable to estimate the number of servings or duration of activity were coded as not meeting that guideline. Sociodemographic variables were sex, age, education, annual household income, years lived in the United States, self-assessed English language proficiency (using a 5-point Likert scale item that included fluent, good, so-so, not very good, not at all), marital status, self-perceived health status (measured by a single question that asked, “In general, would you say your health is. . . ?” with response options of excellent, very good, good, fair, or poor), having a particular place for health care, and having a personal physician.

### Statistical analysis

In 2012 and 2013, we analyzed the preintervention and postintervention survey data by using SAS version 9.3 (SAS Institute, Inc). We compared sociodemographic and health characteristics of participants in each study arm and before-and-after change in knowledge and behavior meeting HEPA guidelines by using linear models. We also estimated unadjusted and adjusted odds ratios (AORs) with 95% confidence intervals (CIs) for the intervention effect and for the change from preintervention to postintervention in each study arm by using logistic regression models with main effects for study arm and time, and a study arm-by-time interaction; adjusted models controlled for participant characteristics and agency. We used generalized estimating equations in all analyses to account for participant clustering by lay health worker and correlation between preintervention and postintervention data on the same person.

## Results

**Figure 2:**
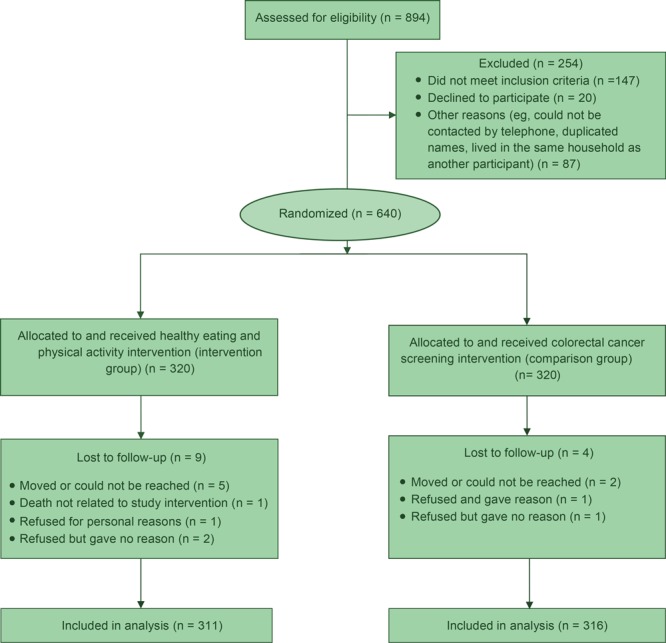
Cluster randomized trial of a lay health worker intervention to increase healthy eating and physical activity among Vietnamese Americans, Santa Clara County, California, 2008–2013.

The 16 lay health workers recruited 894 potential participants, and the study enrolled 640 participants (participation rate, 71.6%). Reasons for exclusion included not meeting inclusion criteria (n = 147) and declining participation (n = 20). The overall 6-month retention rate for the study was 98.0% (627 of 640). Participants lost to follow-up after at least 7 attempts at contact (n = 13) did not complete the follow-up survey (Figure 2).

The 2 groups of participants had similar characteristics, except that HEPA participants were significantly more likely than comparison participants to rate their health as fair, poor, or “don’t know” (Table 1). Each group had equal numbers of men and women by study design.

**Table 1:** Baseline Sociodemographic and Health Characteristics of Vietnamese American Participants Aged 50 to 74 (N = 640) by Study Arm, Santa Clara County, California, 2008–2013^a^

Characteristic	Nutrition and Physical Activity Education Intervention Group (n = 320)	Colorectal Cancer Screening Education Comparison Group (n = 320)	*P* Value^b^
**Female,^c^**	50.0	50.0	>.99
**Age, y**			
50–64	75.0	67.8	.18
65–74	25.0	32.2	
**No. of years in the United States**			
≤10	37.7	39.1	.79
>10	62.3	60.9	
**Self-reported spoken English proficiency**			
Fluent or good	4.4	8.4	.09
So-so	24.8	19.7	
Not very good or not at all	70.9	71.9	
**Education**			
≤Elementary school	19.1	22.4	.60
Junior high or some high school	17.9	22.1	
High school graduate or equivalent	22.9	20.2	
Some college	29.2	25.3	
≥College graduate	11.0	9.9	
**Employment**			
Employed	27.2	26.9	.95
Unemployed, homemaker, student, retired, disabled	72.8	73.1	
**Marital status**			
Married or living with partner	62.2	64.1	.69
Separated, widowed, divorced, never married	37.8	35.9	
**Health insurance**			
None	29.4	30.9	.30
Indigent care from county	16.3	10.9	
Medicare or Medicaid	41.3	42.8	
Private	13.1	15.3	
**Annual household income, $**			
<10,000	15.3	17.8	.14
10,000 to <20,000	24.1	20.3	
20,000 to <40,000	16.3	10.9	
≥40,000	9.7	12.5	
Don’t know or refused	34.7	38.4	
**Self-reported health status**			
Excellent, very good, or good	38.4	49.7	.02
Fair, poor, or don’t know	61.6	50.3	
**Had a particular place for health care**	55.9	61.3	.22
**Had a personal doctor**	69.1	71.2	.64

a Research staff members conducted telephone survey of all participants. Values are percentages unless otherwise indicated.

b *P* values are from linear models using generalized estimating equations to account for clustering of participants by lay health worker.

c Equal numbers of men and women by design.

From preintervention to postintervention, knowledge of the recommended minimum daily number of fruit and vegetable servings increased significantly from 2.6% to 60.5% (*P* < .001) in the HEPA group and from 2.9% to 6.7% (*P* = .006) in the comparison group (Table 2). Knowledge of the physical activity recommendation increased from 2.6% to 62.4% (*P* < .001) in the intervention group and from 1.0% to 2.5% (*P* = .08) in the comparison group.

**Table 2:** Telephone Survey Results on Knowledge and Behaviors Related to HEPA Guidelines^a^ at Preintervention and Postintervention, by Study Group (N = 627), Among Vietnamese Americans Aged 50 to 74 in Santa Clara County, California, 2008–2013^b^

Knowledge or Behavior	HEPA Intervention Group (n = 311)			Colorectal Cancer Screening Comparison Group (n = 316)			*P* Value for Post-Pre Differences Between Groups^c^
	Pre	Post	*P* Value^c^	Pre	Post	*P* Value^c^	
**Knowledge of HEPA guidelines**							
Knows that the recommended minimum daily number of fruit and vegetable servings is 5 per day	2.6 (8)	60.5 (188)	<.001	2.9 (9)	6.7 (21)	.006	<.001
Knows that the recommended minimum weekly amount of moderate and/or vigorous physical activity is 150 minutes per week	2.6 (8)	62.4 (194)	<.001	1.0 (3)	2.5 (8)	.08	<.001
**Self-reported behavior meeting HEPA guidelines**							
Met the guideline for daily fruit and vegetable consumption	8.4 (26)	62.1 (193)	<.001	5.1 (16)	12.7 (40)	.009	<.001
Met the guideline for physical activity	28.9 (90)	54.0 (168)	<.001	38.0 (120)	46.8 (148)	.003	.001

Abbreviation: HEPA, healthy eating and physical activity.

a Fruit and vegetable intake guidelines adapted from *Dietary Guidelines for Americans, 2005* (12). Physical activity guidelines adapted from *Physical Activity Guidelines for Americans, 2008* (13).

b The intervention consisted of 2 educational sessions. Research staff members conducted a single telephone survey 6 months after the first educational session. All values are expressed as percentage (number) unless otherwise indicated.

c *P* values are from linear models using generalized estimating equations to account for clustering of participants by lay health worker.

Self-reported intake of at least 5 servings of fruits and vegetables daily increased significantly in both groups (HEPA group, 8.4% to 62.1%, *P* < .001; comparison group, 5.1% to 12.7%, *P* = .009) (Table 2). Similarly, we found significant increases in the proportion reporting at least 150 minutes of moderate physical activity weekly in both groups: 28.9% to 54.0% (*P* < .001) in the intervention group and 38.0% to 46.8% (*P* = .003) in the comparison group. Increases in knowledge of recommended daily fruit and vegetable intake and of recommended physical activity (both *P* < .001) were significantly greater in the HEPA group than in the comparison group. The HEPA group had significantly greater increases than the comparison group in meeting guidelines for daily fruit and vegetable intake (*P* < .001) and physical activity level (*P* = .001).

After we adjusted for sociodemographic and health characteristics, participants in the HEPA group had a greater increase than did those in the comparison group in the odds of knowing the recommended minimum daily number of fruit and vegetable servings (AOR = 39.0; 95% CI, 11.6–131.2) and weekly physical activity recommendation (AOR = 47.4; 95% CI, 9.1–245.8) (Table 3). Furthermore, the intervention group had a greater increase than the comparison group in the odds of meeting the daily fruit and vegetable intake recommendation (AOR = 8.8; 95% CI, 3.8–20.3) and the weekly physical activity recommendation (AOR = 2.2; 95% CI, 1.4–3.6).

**Table 3:** Intervention Effects on Knowledge and Behaviors Related to HEPA Guidelines^a^ Among Vietnamese Americans Aged 50 to 74 (N = 627) in Santa Clara County, California, 2008–2013^b^

Effect	Knowledge of HEPA Guidelines		Behavior Meeting HEPA Guidelines	
	Daily Fruit And Vegetable Intake	Weekly Physical Activity	Self-Reported Daily Fruit And Vegetable Intake	Self-Reported Weekly Physical Activity
**Unadjusted model**				
Intervention effect^c^	23.7 (7.9–71.5)	23.4 (5.9–93.5)	6.6 (3.0–14.5)	2.0 (1.3–3.1)
Postintervention vs preintervention (effect of time) in comparison group^d^	2.4 (1.3–4.5)	2.7 (1.0–7.5)	2.7 (1.5–4.9)	1.4 (1.1–1.8)
Postintervention vs preintervention (effect of time) in intervention group^d^	57.7 (23.1–143.9)	63.2 (24.7–161.2)	17.9 (10.6–30.3)	2.9 (2.0–4.1)
**Adjusted multivariable model**				
Intervention effect^c^	39.0 (11.6–131.2)	47.4 (9.1–245.8)	8.8 (3.8–20.3)	2.2 (1.4–3.6)
Postintervention vs preintervention (effect of time) in comparison group^d^	2.5 (1.3–4.5)	2.4 (0.8–7.5)	2.7 (1.5–4.7)	1.5 (1.1–1.9)
Postintervention vs preintervention (effect of time) in intervention group^d^	95.5 (34.3–266.0)	113.6 (36.0–358.3)	23.4 (12.6–43.2)	3.2 (2.2–4.8)

Abbreviation: HEPA, healthy eating and physical activity.

a Fruit and vegetable intake guidelines adapted from *Dietary Guidelines for Americans, 2005* (12). Physical activity guidelines adapted from *Physical Activity Guidelines for Americans, 2008* (13).

b All logistic regression models accounted for lay health worker clustering using generalized estimating equations, and multivariable models were adjusted for additional covariates: agency, age, sex, highest education level, household income, self-reported spoken English proficiency, years in the US, marital status, self-reported health status, having a usual source of health care, and having a personal physician. Values expressed are odds ratio (95% confidence interval); 18 participants had missing data for 1 or more covariates and were excluded from the multivariable models.

c The increase in the odds of knowing the guideline or in meeting the guideline for HEPA group participants compared with those in the comparison group. Intervention-arm participants attended 2 educational sessions about HEPA. Comparison-arm participants attended 2 educational sessions about colorectal cancer screening.

d The increase in the odds of knowing the guideline or in meeting the guideline for participants within the same group preintervention and postintervention.

## Discussion

This study is the first randomized controlled trial to evaluate the efficacy of a lay health worker–delivered HEPA educational intervention among Vietnamese Americans, and among the few randomized controlled trials of HEPA interventions among Asian Americans. In our sample of older, limited–English-proficient Vietnamese Americans, those who received the lay health worker HEPA educational intervention had significant increases in both HEPA knowledge and self-reported behaviors meeting recommended guidelines, and these increases were greater than increases in the comparison group. Our study not only adds to evidence of effectiveness of lay health worker interventions but is among the first to address HEPA behaviors, which can be difficult to change, among Asian Americans.

Baseline rates of knowledge and self-reported adherence to recommendations for fruit and vegetable intake were low among Vietnamese Americans; this finding is consistent with published studies of other racial/ethnic groups (25,26). A study published almost 25 years ago reported that Vietnamese Americans in California consumed only 3.5 servings of fruits and vegetables per day (8). Only about half of participants in our study could estimate the number of servings of fruits and vegetables per day eaten at baseline, so we do not have a good estimate of the average number of servings in our study population. Nationally, the median daily intake of fruits is 1.0 time daily and of vegetables is 1.7 times daily (27). Much work remains to address suboptimal levels of fruit and vegetable intake among Americans in general but particularly among underserved populations, including Vietnamese Americans.

Among HEPA intervention participants, the lay health worker–delivered intervention led to increases of 57.9 and 53.7 percentage points, respectively, in the proportion of those knowing the recommended daily fruit and vegetable intake and of those meeting daily recommended fruit and vegetable intake. Our findings are similar to those in other studies showing favorable increases in nutrition knowledge and behavior among at-risk adults attending interactive nutrition-focused educational sessions (26,28,29). In addition to the 2 lay health worker educational sessions, HEPA participants also received a bilingual booklet with messages and images similar to those presented in the small group sessions and, among those identified as not meeting recommended HEPA guidelines, also received additional telephone-based support from lay health workers to identify barriers and to provide suggestions to overcome barriers to HEPA behaviors. Future research could examine which elements of this lay health worker intervention are most effective in promoting healthy eating knowledge and behavior change.

At baseline, few (2.6%) Vietnamese Americans in our study knew the recommended level of physical activity. This baseline rate is similar to that found in another study (2.8%), in which Chinese Americans received print materials and educational lectures (26). Our lay health worker intervention was effective in increasing knowledge of the recommended level of physical activity by almost 60 percentage points in the intervention group, whereas knowledge in the comparison group increased by only 1.5 percentage points.

Although baseline rates of knowledge of the recommended level of physical activity were low in our study population, about one-third reported physical activity that met the minimum recommended level of physical activity. This baseline level of meeting physical activity recommendations is lower than that found in another study reporting that 55% of older Chinese Americans met this recommendation (26). Postintervention, we found that self-reported physical activity meeting recommendations increased significantly by 25 percentage points among intervention group participants and significantly by 8.8 percentage points among comparison group participants. A meta-analysis of physical activity interventions reported that behavioral strategies (eg, goal setting) are more effective than cognitive strategies (eg, health education) to increase physical activity (30). This result suggests that the telephone-based support provided by the lay health worker might have been an essential component to increase reported physical activity in our study population.

The large odds ratios with wide confidence intervals for intervention effects on knowledge and adherence to guidelines on fruit and vegetable intake resulted from low levels at baseline followed by large increases in the intervention group (who were taught the guidelines) but not in the comparison group. The small numbers also resulted in large standard errors of log odds ratio estimates, which were magnified in the confidence intervals for odds ratios. After covariate adjustment, the estimates and standard errors of intervention effects increased, leading to more extreme odds ratios with larger confidence intervals but not changing the overall findings.

Our study had several limitations. First, we used self-reported data on adherence, which could be subject to overestimation by participants. This overestimation might be due to misperception of the serving sizes for fruits and vegetables (31), including the challenges in accurately estimating serving sizes of fruits and vegetables among Asians, who in general may prefer family-style eating (24). It is also possible that baseline and comparison group fruit and vegetable consumption was underestimated — only about half the participants were able to report the number of servings consumed at baseline. In addition, self-reported fruit and vegetable consumption on the previous day may not reflect regular patterns of consumption. Second, this study incorporated national dietary guidelines that were current when the study was initiated (12) but are no longer the norm. Third, our randomized controlled trial did not include a no-intervention control group; the slight increases found in the comparison group could be attributed to increased exposure to information through repeated surveys or to secular trends. Finally, our findings may not be generalizable to other Vietnamese American communities in the United States. On the other hand, the strengths of this study include strong stakeholder engagement, a large sample size, and a high retention rate in an understudied population.

Our study adds to the limited literature examining HEPA knowledge and behaviors among Asian Americans and particularly among Vietnamese Americans, a growing population that is underserved in health education, health intervention, and social programs. With a large study sample of Vietnamese Americans, we demonstrated the efficacy of a lay health worker intervention to increase both HEPA knowledge and self-reported behaviors. Our study showed significant increases in knowledge and behaviors: 50% to 60% of intervention participants increased their HEPA knowledge or reported increases in HEPA behaviors meeting recommended guidelines, but more research is needed to understand how to increase these proportions. Additional future directions include examining how and which participants respond to which elements of the lay health worker intervention. Regardless, a lay health worker–led intervention is a feasible, accessible, and efficacious approach to addressing disparities in HEPA knowledge and behaviors and to promoting health equity in this population.
